# Tribological Behavior of Reduced Graphene Oxide–Al_2_O_3_ Nanofluid: Interaction among Testing Force, Rotational Speed and Nanoparticle Concentration

**DOI:** 10.3390/ma15155177

**Published:** 2022-07-26

**Authors:** Chenglong Wang, Jianlin Sun, Linghui Kong, Jiaqi He

**Affiliations:** 1 School of Materials Science and Engineering, University of Science and Technology Beijing, Beijing 100083, China; ustbwcl@163.com (C.W.); ustbhjq@163.com (J.H.); 2 The Yellow River Institute of Hydraulic Research, the Yellow River Water Conservancy Commission, Zhengzhou 450003, China

**Keywords:** reduced graphene oxide, tribology, nanofluid, lubrication mechanism, response surface methodology

## Abstract

The tribological properties of nanofluids are influenced by multiple factors, and the interrelationships among the factors are deserving of further attention. In this paper, response surface methodology (RSM) was used to study the tribological behavior of reduced graphene oxide–Al_2_O_3_ (rGO-Al_2_O_3_) nanofluid. The interaction effects of testing force, rotational speed and nanoparticle concentration on the friction coefficient (*μ*), wear rate (*W*_r_) and surface roughness (*R*_a_) of steel disks were investigated via the analysis of variance. It was confirmed that all the three input variables were significant for *μ* and *W*_r_ values, while testing force, nanoparticle concentration and its interaction with testing force and rotational speed were identified as significant parameters for *R*_a_ value. According to regression quadratic models, the optimized response values were 0.088, 2.35 × 10^−7^ mm^3^·N^−1^·m^−1^ and 0.832 μm for *μ*, *W*_r_ and *R*_a_, which were in good agreement with the actual validation experiment values. The tribological results show that 0.20% was the optimum mass concentration which exhibited excellent lubrication performance. Compared to the base fluid, *μ*, *W*_r_ and *R*_a_ values had a reduction of approximately 45.6%, 90.3% and 56.0%. Tribochemical reactions occurred during the friction process, and a tribofilm with a thickness of approximately 20 nm was generated on the worn surface, consisting of nanoparticle fragments (rGO and Al_2_O_3_) and metal oxides (Fe_2_O_3_ and FeO) with self-lubrication properties.

## 1. Introduction

During material manufacturing and processing, appropriate lubrication techniques are required to reduce friction and wear. Lubricants are critical for cutting [1], rolling [2], forging [3] and other metal forming processes to provide antiwear, cooling and cleaning effects. The application of lubrication can decrease energy consumption, increase productivity and improve the surface quality of products [4,5].

In recent years, with the development of nanotechnology, nanofluids with excellent thermal conductivity, chemical stability and lubricating properties have attracted extensive attention and investigation [6]. For example, thermal transport was significantly enhanced by adding Fe_3_O_4_-CuO [7] and TiO_2_ [8] nanoparticles to water-based fluids. Imran [9] and Muhammad et al. [10] numerically investigated the two-dimensional flow and nonlinear thermal radiation of nanoparticles to reveal the interrelationship between temperature distribution and heat transport mechanisms. It has been documented that reduced graphene (rGO) and aluminum oxide (Al_2_O_3_) nanoparticles as lubricant additives exhibit favorable friction-reducing and antiwear properties under various conditions [11,12]. Gupta et al. [13] investigated the lubrication mechanism of rGO nanosheet in polyethylene glycol. An extremely low concentration (0.02 mg/mL) of rGO nanoparticle was adequate to effectively lubricate the steel-steel sliding surface, which was attributed to the deposition of rGO in the contact area and the formation of a protective tribofilm. He et al. [14] studied the lubrication performance of nano-Al_2_O_3_ in water-based fluids using a ball-on-three-plate tester. The results show that Al_2_O_3_ nanoparticles in water embedded into the steel substrate and acted as the load bearer during the friction process. Research has revealed that rGO-Al_2_O_3_ nanofluid exhibited good dispersion stability [15]. In addition, the combination of layered rGO with an outstanding antifriction effect and spherical Al_2_O_3_ with high extreme pressure properties showed a synergistic lubricating effect [16]. However, numerous factors influence the tribological properties of nanofluids, such as concentration, rotational speed of friction pairs and test force. The study of these independent factors and the interaction between factors are significant for optimizing the combination of process parameters in actual metal processing, predicting the effect of parameter variations on metal surface quality and revealing the lubrication mechanism of nanofluids. Response surface methodology (RSM) is a mathematical and statistic method, which is helpful for analyzing the effect of input variables and their interaction on response results. Researchers [17] investigated the effect of turning conditions and cutting fluid concentration on cutting force and surface roughness during the turning process. Regression models were developed, and the optimum input variables were obtained. In addition, the pin-on-disk tribological test has been widely used to evaluate the frictional lubrication properties of nanofluids [18,19]. The tribological property of graphene as an additive in canola oil was investigated through a pin-on-disk tribometer by Omrani et al. [19]. The lowest friction coefficient and wear rate were observed when the concentration of graphene was 0.7 wt.%. More importantly, the study found that it was the solid lubricant rather than the oil which exerted a lubricating effect on the pin surface. He et al. [20] studied the tribological behavior of hexagonal boron nitride (h-BN) nanofluids under different testing forces. The results show that the friction coefficient decreased with the increase in the testing force, and the surface quality of the steel disks was improved by the polishing effect of nanoparticles. Therefore, the tribological behavior of nanofluids investigated via pin-on-disk experiments was of great importance to reveal the lubrication mechanism. Combining the RSM to reveal the interaction rather than individual effects of various tribological parameters was also a research focus.

In this study, rGO-Al_2_O_3_ nanofluids of different concentrations were prepared. The tribological behavior was studied on a pin-on-disk tribometer, and the synergistic lubrication mechanism was revealed. Furthermore, RSM method was employed to design subsequent tests, and regression quadratic models were gained to analyze the effect of testing force, rotational speed and nanoparticle concentration on friction coefficient, wear rate and worn surface roughness. Finally, optimized input and response values were obtained through the above models, then validation tests were conducted to verify their accuracy. By investigating the interrelationship between the tribological properties of nanofluids and various factors, the corresponding quantitative mathematical models were established, which can provide some theoretical support for the setting of process parameters for future metal working processes.

## 2. Materials and Methods

### 2.1. Materials

GO (≥98%) nanosheet with a thickness of 5–10 nm and the diameter of 3–5 μm was obtained from Shandong OBO New Materials Co., Ltd. (Dongying, China). The specific parameters of GO are listed in Table 1. Aluminum isopropoxide (AI, AR) was purchased from Macklin Biochemical, Co., Ltd. (Shanghai, China). Organic molybdenum (OM) was acquired from Suzhou Jinmu Runcheng Lubrication Technology Co., Ltd.(Suzhou, China). Glycerol (>98%), triethanolamine (TEA, >98%), sodium hexametaphosphate (SHMP, CP) and sodium dodecyl benzene sulfonate (SDBS, AR) were provided by Sinopharm Chemical Reagent Co., Ltd. (Shanghai, China). All the reagents were used as received without further purification in this research.

### 2.2. Preparation of Nanofluids

In our preliminary study [16], rGO-Al_2_O_3_ nanocomposite was synthesized through a hydrothermal method. Briefly, 0.1 g of GO powder was added to 49.9 g of deionized water and stirred for 1 h. Then, 0.2 g of AI was added to 49.8 g of deionized water, heated to 90 °C and homogeneously stirred. The two aqueous solutions were blended and regulated to be weakly alkaline (pH = 8) using TEA. Next, the mixing solutions were transferred to a reactor, heated to 210 °C and maintained for 12 h to acquire a black colloid. The obtained colloid was freeze-dried after several centrifugations, filtration and rinsing. Finally, the rGO-Al_2_O_3_ nanocomposite was fabricated using a heat treatment at 500 °C for 8 h. rGO-Al_2_O_3_ nanofluids with different nanoparticle concentrations (0.1, 0.2 and 0.3 wt.%) were prepared by adding rGO-Al_2_O_3_ nanocomposite, 2.5 wt.% OM, 2.5 wt.% glycerol, 2.0 wt.% TEA and 0.3 wt.% SDBS in deionized water. OM was used as an extreme pressure agent to improve the load-bearing capacity of the nanofluids. Glycerol and TEA were used as a lubrication additive and pH regulator, respectively. SDBS was used as a dispersant and surfactant to improve the dispersion stability of nanofluids. In addition, base fluid was prepared without the rGO-Al_2_O_3_ nanocomposite as the control group, and the physicochemical properties of the base fluid are listed in Table 2. This paper mainly investigated the influence of nanoparticles on tribological behavior. Therefore, the composition and content of the base fluid remained constant, and all nanofluids differed only in the concentration of nanoparticles, to avoid the influence of important factors such as rheological properties, viscosity, fluid film and the surface separation of nanofluids on tribological properties.

### 2.3. Experimental Design

**Figure 1:**
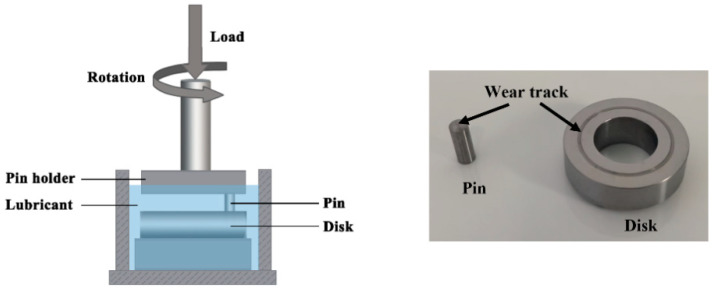
Schematic diagram of the disk-on-disk tribotester and the photo of the pin and disk.

The tribological behavior of rGO-Al_2_O_3_ nanofluids were assessed using an MM-W1A pin-on-disk, and the schematic diagram is illustrated in Figure 1. The pin and disk were made of 1045 steel, and the relevant chemical compositions are listed in Table 3. The steel disk had strict machining accuracy requirements (the upper surface roughness needed to be 0.4 μm), and a uniform polishing process was performed before the tests. According to ASTM G99-2016, the friction coefficient (μ) of the steel/steel friction pairs was obtained at the speed of 200–400 rpm and the load of 100–300 N. Our original intention in designing this experiment was to analogize it to the rolling process. In our previous study [16], the rolling force of steel cold rolling was in the range of 50–200 kN, and the contact surface between the strip and roll was 355.59–377.97 mm^2^, so the contact pressure was in the range of 132–562 MPa. In addition, during the pin-on-disk friction process, the contact surface between the pin and disk was about 0.442 mm^2^. To simulate the pressure of the actual rolling process, the test force should in the range of 58–248 N. As a result, the pin was loaded at 100 N, 200 N and 300 N in our study. Similarly, the test speed was also chosen considering the actual material processing. We used an experimental mill with a speed of 30–60 rpm and a radius of 130 mm. In order to keep the linear speed consistent with the tribological experiments, the speed of the pin-on-disk tribotester was kept at 163–325 rpm. The experimental time was set to 30 min, and each group of tests was performed three times to minimize experimental error. During the tribological experiments, the temperature and other factors were kept as stable as possible to ensure the reliability of the data. Ra value was the most commonly discussed of all roughness parameters and was applied to describe the quality of the worn surface [21]. Surface topography and roughness of the wear track on disks were characterized using a laser scanning confocal microscope (LSCM, Olympus LEXTOLS 4100, Tokyo, Japan).The wear rate (*W*_r_) of the disk was established using the weight-loss method and specified as [22]:

(1) $$W_{r}=\frac{\Delta m}{\rho \cdot F\cdot l}$$

where Δm, ρ, *F* and *l* represent the wear weight loss, density of the disk, normal force and total wear stroke.

In order to investigate the effect of independent variables, the testing force (*F*), rotational speed of the disk (*V*) and nanoparticle concentration (*N*_c_) on tribological performance, a mathematical model based on response surface methodology (RSM) was developed. The *μ* value of fluids and the *W*_r_ and *R*_a_ values of wear track on steel disks were chosen as responses in this study. Three levels were defined for each variable using the Box–Behnken design as shown in Table 4. A total of 15 trials were conducted according to the design. All the tests were carried out in triplicate, and the average values were employed as response. A quadratic model was used to establish the relationship between these three variables and responses, which can be defined as Equation (2).

(2) $$Y=A_{o}+\overset{n}{\underset{i=1}{\sum }}A_{i}X_{i}+\overset{n}{\underset{i=1}{\sum }}A_{i}X_{i}^{2}+\overset{n}{\underset{i<j}{\sum }}A_{ij}X_{i}X_{j}$$

where *Y* is the desired response; *A*_0_ is a constant; and *A_i_*, *A_j_* and *A_ij_* are coefficients of linear, quadratic and cross-product terms, respectively. *X_i_* represents the input variables corresponding to the friction process.

Design Expert software (version 13.0, Beijing, China) was employed for the regression, fitting process, the acquirement of 3D response surface and optimal response. The analysis of variance (ANOVA), the coefficient of determination (R^2^) and adjusted R^2^ were applied to verify the goodness of fit for the obtained models through the significance of regression and individual model coefficient.

### 2.4. Sample Characterization and Analysis of Worn Surface

Transmission electron microscopy (TEM, JEOL JEM-2010, Tokyo, Japan) was adopted to study the morphology and structure of different nanoparticles. The crystal structure and chemical composition of the rGO-Al_2_O_3_ nanoparticle were characterized using X-ray photoelectron spectroscopy (XPS, Kratos AXIS Ultra, Manchester, UK). X-ray powder diffraction (XRD, Rigaku Ultima IV, Tokyo, Japan) was conducted at 2θ range of 10~60°. The wear morphologies on the steel disk surface were observed using a laser scanning confocal microscope (LSCM, Olympus LEXT OLS4100, Tokyo, Japan). The worn surface of the steel disk was investigated using a scanning electron microscope (SEM, ZEISS Sigma 500, Oberkochen, Germany) with an energy dispersive spectrometer (EDS). The cross section of the steel disk was acquired using a focused ion beam microscope (FIB, Helios NanoLab 600i, Hillsboro, OR, USA). Subsequently, the morphology and chemical composition of the tribofilm were further characterized through TEM and XPS, to propose the lubrication mechanism of the rGO-Al_2_O_3_ nanofluid.

## 3. Results and Discussion

### 3.1. Microstructure and Composition of rGO-Al_2_O_3_ Nanoparticle

**Figure 2:**
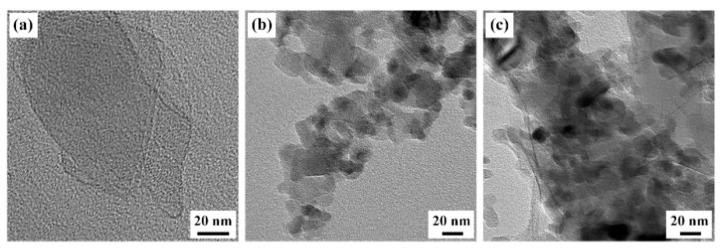
TEM images of (**a**) rGO, (**b**) Al_2_O_3_ and (**c**) rGO-Al_2_O_3_ nanoparticles.

The morphologies of rGO, Al_2_O_3_ and rGO-Al_2_O_3_ nanoparticles were characterized using TEM, as presented in Figure 2. In Figure 2a, the rGO nanoparticle consisting of several stacked lamellae, exhibited a smooth surface and large lateral dimensions. Whereas the Al_2_O_3_ nanoparticle was easily agglomerated and freely associated with other Al_2_O_3_ nanoparticles, thereby enhancing the particle size. For the synthesized Al_2_O_3_ nanoparticle, as shown in Figure 2c, it was clearly seen that the Al_2_O_3_ nanoparticle was randomly distributed on the lamellae of rGO. The diameter of nano-Al_2_O_3_ was less than 20 nm, and there was no obvious agglomeration, although the edges of the rGO nanosheet appeared to be slightly folded.

**Figure 3:**
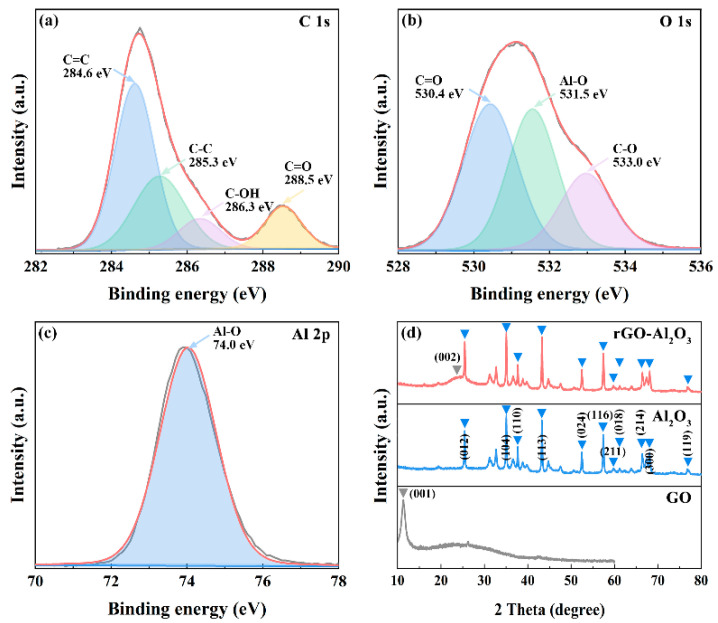
(**a**) C 1s, (**b**) O 1s, (**c**) Al 2p XPS spectra of the rGO-Al_2_O_3_ nanocomposite, and (**d**) XRD patterns of different nanoparticles.

The XPS spectra in Figure 3 were employed to investigate the compositions and chemical states of the rGO-Al_2_O_3_ nanoparticle. The C 1s peaks in Figure 3a were located at 284.6 eV, 284.6 eV, 284.6 eV and 284.6 eV, corresponding to the C=C, C-C, C-OH and C=O function groups [16]. The intensities of C-OH and C=O were weaker than those of C=C and C-C, demonstrating that GO was reduced in the hydrothermal reaction [15]. The O 1s spectrum was divided into three peaks at 530.4 eV, 531.5 eV and 533.0 eV associating with C=O, Al-O and C-O, respectively. Coupled with Al-O at 74.0 eV of the Al 2p spectrum in Figure 3c, the presence of nano-Al_2_O_3_ in the nanocomposite was confirmed. The XRD patterns of different nanoparticles are shown in Figure 3d. The characteristic peaks of nano-Al_2_O_3_ recorded at 25.6°, 35.1°, 37.8°, 43.4°, 52.6°, 57.5°, 59.8°, 61.3°, 66.5°, 68.2° and 77.2° related to the (012), (104), (110), (113), (024), (116), (211), (018), (214), (300) and (119) planes of α-Al_2_O_3_ (JCPDS card No.10-0173). These diffraction peaks were also found in the rGO-Al_2_O_3_ nanocomposite. It is noteworthy that the sharp peak (001) of GO was absent in the rGO-Al_2_O_3_ nanocomposite, while a new diffraction peak (corresponding to the (002) plane of rGO) was observed at the position of 24.4°. This was sufficient to demonstrate that GO was reduced to rGO and combined with Al_2_O_3_. Associated with the results of XPS and XRD, the successful synthesis of the rGO-Al_2_O_3_ nanocomposite was evidenced.

### 3.2. Tribological Behavior Analysis Based on the RSM Method

#### 3.2.1. Experimental Results

The pin-on-disk tribological tests of rGO-Al_2_O_3_ nanofluids were conducted as per the design illustrated in Table 5, and the output in terms of the three response values were listed. The obtained friction coefficient (*μ*), wear rate (*W*_r_, calculated by Equation (1)) and surface roughness (*R*_a_) were imported into the Design Expert 13.0 software for the subsequent data analysis. The analysis of the variance (ANOVA) of response results was carried out with the objective of analyzing the influence of test conditions and nanoparticle concentration on the obtained results. Table 6, Table 7 and Table 8 show the ANOVA results of *μ*, *W*_r_ and *R*a values, respectively. These analyses were carried out at the significance level of 5% meaning that when the *p*-values were less than 0.05 (or 95% confidence), the corresponding factors were considered to be statistically significant to the response value. Table 6 and Table 7 show that all three factors had significant influence on the *μ* and *W*_r_ values of the nanofluids. Testing force (*F*), nanoparticle concentration (*N*_c_) and its interaction with speed (*V*) were significant to the Ra value of the steel disk, as illustrated in Table 8.

**Figure 4:**
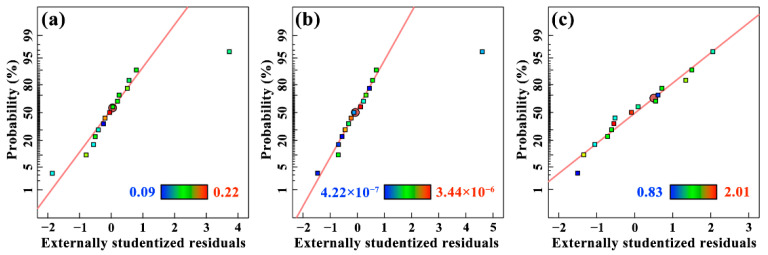
Normal probability plot of residual for (**a**) *μ*, (**b**) *W*_r_ and (**c**) *R*_a_.

The images in Figure 4 are externally studentized residuals of the three response values. It was obvious that all the data closely followed a straight line, which indicated that the data distribution law was normal [23]. It further demonstrated that the models proposed were adequate and reasonable. In addition, this judgement can also be derived from Table 6, Table 7 and Table 8 as the lack of fit items was not significant. 

#### 3.2.2. Quadratic Models and Response Surface Analysis

**Figure 5:**
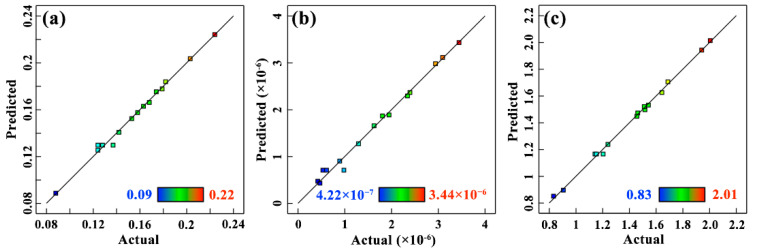
The parity plot illustrating the correlation between actual and predicted values for (**a**) *μ*, (**b**) *W*_r_ and (**c**) *R*_a_.

The initial analysis of the response values obtained from RSM included all input variables and their interactions. The regression models obtained according to the quadratic model for the *μ*, *W*_r_ and *R*_a_ response values are given in Equations (3)–(5). According to Equation (3), the coefficient of *F* was positive and the coefficient of *V* and *N*_c_ was negative, indicating that the *μ* value increased with the increase in testing force and decreased with the increase in nanoparticle concentration and speed. Whereas the coefficient of the secondary term *N*_c_^2^, which also had a significant influence, was positive, indicating that the *μ* value decreased first and then increased with the increase in concentration. As a result, there was a value of optimal concentration in the range of 0.1~0.3 wt.%. Similarly, according to Equations (4) and (5), the influence of concentration on *W*_r_ and *R*_a_ was consistent with its relationship with the *μ* value. With regards to the influencing factors of wear rate, the primary term (*F*, *V* and *N*_c_) and the quadratic term (*F*^2^, *V*^2^ and *N*_c_^2^) were significant; however, the positive and negative values of the coefficients (in the primary term and the quadratic term) were completely opposite. Therefore, the influence of these factors on *W*_r_ need to be further analyzed using the 3D response surface plots. The coefficient of determination (R^2^) and adjusted R^2^ of these three values were 0.9937, 0.9908, 0.9975 and 0.9824, 0.9743, 0.9930, respectively. These values are much higher than the results of the previous studies by Bouacha [23] and Sharma et al. [17]. Furthermore, Figure 5 shows the parity plots between the actual and predicted values, and the points clustered near to the diagonal line of the plot indicate that the predicted data by models were very close to the actual test results [24]. Hence, it was concluded that these quadratic mathematical models are highly accurate and persuasive and can be used to analyze the effect of input variables on response results to obtain optimal solutions.

(3) $$\begin{array}{l} \mu =0.469+0.0002\cdot F-0.000342\cdot V-2.55292\cdot N_{c}-6.25\times 10^{-7}\cdot F\cdot V+0.0002\cdot F\cdot N_{c}+\\ 0.000925\cdot V\cdot N_{c}-4.33\times 10^{-7}\cdot F^{2}+1.42\times 10^{-7}\cdot V^{2}+5.31667\cdot N_{c}^{2} \end{array}$$

(4) $$W_{r}=5.89\times 10^{-6}-5.44\times 10^{-9}\cdot F-1.52\times 10^{-8}\cdot V-0.000033\cdot N_{c}-1.61\times 10^{-11}\cdot F\cdot V-1.26\times 10^{-9}\cdot \;F\cdot N_{c}-6.70\times 10^{-9}\cdot V\cdot N_{c}+5.37\times 10^{-11}\cdot F^{2}+3.00\times 10^{-11}\cdot V^{2}+0.000094\cdot N_{c}^{2}({\mathrm{mm}}^{3}\;N^{-1}\;m^{-1})\;\;\;\;\;\;\;\;\;\;\;\;\;\;$$

(5) $$R_{a}=1.55313+0.002041\cdot F+0.002786\cdot V-13.535\cdot N_{c}+1.70\times 10^{-6}\cdot F\cdot V-0.007025\cdot F\cdot \;N_{c}-0.008175\cdot V\cdot N_{c}+5.08\times 10^{-6}\cdot F^{2}-2.58\times 10^{-6}\cdot V^{2}+44.35\cdot N_{c}^{2}\;(\mu m)\;\;\;\;\;\;\;\;\;\;\;\;\;\;\;\;\;\;\;\;\;\;\;\;\;\;\;\;\;\;\;\;\;\;\;$$

**Figure 6:**
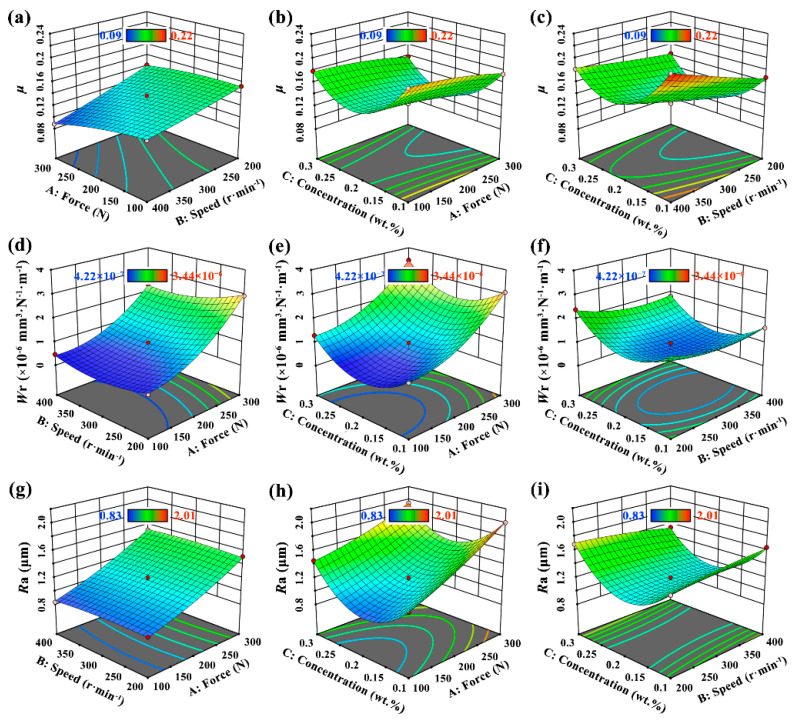
3D response surface plots showing the interaction effects of different variables on (**a**–**c**) *μ*, (**d**–**f**) *W*_r_ and (**g**–**i**) *R*_a_ values.

To intuitively investigate the interaction effects of experimental variables including A-*F*, B-*V* and C-*N_c_* with the response values (*μ*, *W*_r_ and *R*_a_), 3D response surface plots with contour lines were obtained as shown in Figure 6. At one time, two of them were flexible within the experimental ranges, while the others were kept constant at the middle level. The change in the shape of the surface plots reflects the interaction between input variables. It can be seen from Figure 6a–c that a higher testing force and rotation speed contributed to lower *μ* value. For the purpose of reducing friction coefficient, the optimal concentration of rGO-Al_2_O_3_ nanofluid was about 0.20 wt.%. From Figure 6d–f, the lowest *W*_r_ value was obtained using the combination of the lowest testing force and the appropriate speed (about 350 rpm) at the optimal concentration of about 0.19 wt.%. Regarding surface roughness, it can be deduced from Figure 6g–i that the lowest *R*_a_ value was achieved with the lowest testing force and an optimum concentration of about 0.20 wt.%. In the studies of Du [25] and He et al. [26], the best tribological performance was achieved when the nanofluid concentration was 0.5 wt.% and 2.0 wt.%, respectively. In contrast, in our study, the optimum tribological performance was obtained by only adding 0.2 wt.% of rGO-Al_2_O_3_ nanoparticles, which had a significant advantage.

Through the analysis and discussion above, it is worth mentioning that as the nanoparticle concentration increased above 0.20 wt.%, the *μ*, *W*_r_ and *R*_a_ values all became higher, which indicated that the antiwear and antifriction performance of nanofluids were weakened. The general reasons for this phenomenon are as follows. As the nanoparticle concentration increased, the stability of the nanofluids in the process of friction was destroyed, so the nanoparticles were more likely to agglomerate and therefore the μ value rose. This was consistent with the results of our previous study [16]. At the same time, the defect degree of nanoparticles increased after friction, which was also one of the reasons for the deterioration of the lubrication effect with the progress of the friction process. In addition, the abrasive wear caused by agglomerated large-size nanoparticles led to the sharp rise in *W*_r_ and *R*_a_ values [27]. Furthermore, for the *μ* and *W*_r_ values, the change in testing force had a contrasting opposite effect. The friction coefficient was negatively correlated with the testing force, which is contrary to the conventional tribological lubrication process [28]. This is mainly due to the fact that with the increase in pressure during friction, the rolling effect, interlayer sliding effect and polishing effect became stronger, as did the antifriction effect of the nanofluids. This was based on our conclusions derived from simulating the motion of nanoparticles using nonequilibrium molecular dynamics [29]. The movement form of MoS_2_-Al_2_O_3_ nanoparticles during the friction process was reproduced, and it was found that layered MoS_2_ and spherical Al_2_O_3_ occurred during interlayer sliding and rolling, transferring the friction of the Fe surface to the internal friction of nanoparticles, thus playing the role of antifriction [29]. However, the increase in force can increase the wear rate, thus the *W*_r_ value was positively correlated with the testing force. However, it was difficult to control conditions such as velocity and force to achieve the optimal tribological performance in the actual metalworking processes, which creates challenges for the application of the research results. In the early stages of our research, we tried our best to control the force and velocity conditions to keep them close to the actual parameters, hoping to provide theoretical guidance for the actual production process.

#### 3.2.3. Response Optimization

Response surface optimization was helpful to identify the combination of input variables (pin-on-disk test parameters and nanoparticle concentration) that jointly optimize the *μ*, *W*_r_ and *R*_a_ values to a minimum during the friction process. RSM optimization results with predicted and validation results for different response parameters are shown in Table 9. The desirability on a range from 0 to 1 is used to measure the optimization achievement, and the closer this parameter is to 1, the better and more ideal the optimization result [30]. Every validation test was conducted three times.

As shown in Table 9, the differences between the validation and predicted values were only 1.1%, 4.7% and 2.2% for *μ*, *W*_r_ and *R*_a_ values, respectively. Hence, it revealed that the quadratic models of RSM analysis were reliable for predicting experimental results.

### 3.3. Pin-on-Disk Tribological Behavior of Nanofluids

**Figure 7:**
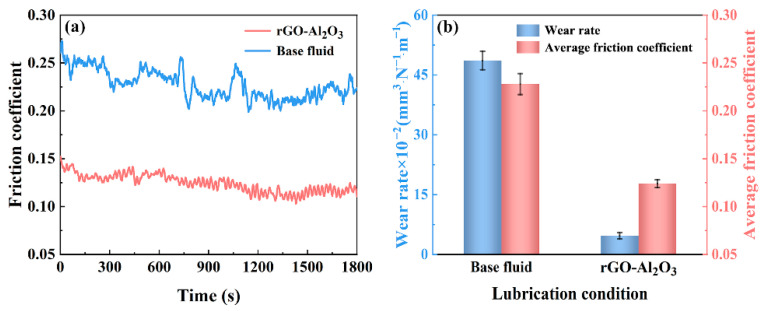
Tribological behavior of base fluid and rGO-Al_2_O_3_ nanofluid: (**a**) friction coefficient–time curves and (**b**) wear rate and average friction coefficient.

The friction coefficient–time curves, average friction coefficient and wear rate of the disk lubricated with base fluid and 0.20 wt.% rGO-Al_2_O_3_ nanofluid are presented in Figure 7. Compared to the base fluid, the average friction coefficient and wear rate of 0.20 wt.% rGO-Al_2_O_3_ nanofluid decreased by about 45.6% and 90.3%, respectively. It appears that the introduction of nanoparticles into the base fluid was effective to reinforce the antiwear and antifriction behavior of lubricants. As seen in Figure 7a, the friction coefficient–time curves of 0.20 wt.% rGO-Al_2_O_3_ nanofluid were relatively smooth and steady without an obvious rise. The curve kept rising and fluctuating for the base fluid: it was zigzagging and unstable throughout the test. The reduction and stability of the friction coefficient was considered to be attributed to the tribofilm formed by nanoparticles [26]. Furthermore, the addition of rGO-Al_2_O_3_ in the base fluid improved the tribofilm stability and strength generated on the surface of friction pairs. This is consistent with the results of previous studies [16,25]. In tribological experiments, the lubrication state of nanofluids generally belongs to boundary lubrication. In the friction contact area, some additives and water were extruded, and nanoparticles mainly played the lubrication role, which was similar to solid lubrication. However, the variation of tribological properties caused by the rheological properties of nanofluids with different concentrations should not be ignored as well.

**Figure 8:**
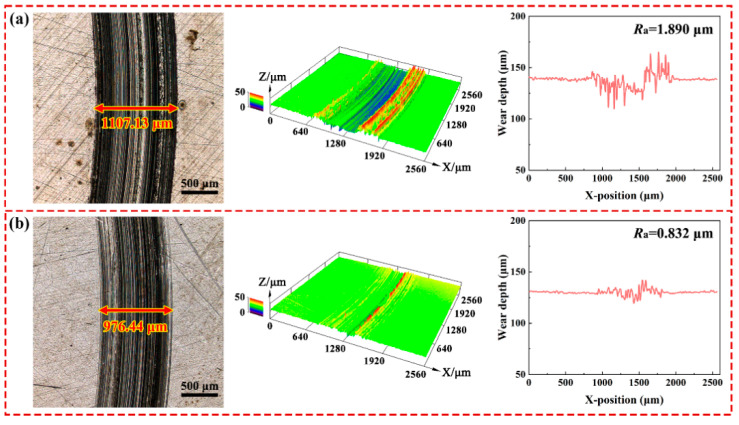
Surface topography and cross-section depth profile of wear track on the disk lubricated by (**a**) base fluid and (**b**) rGO-Al_2_O_3_ nanofluid.

Figure 8 shows the 2D optical images, 3D topographies and cross-section depth profile of wear tracks to evaluate the lubrication effect of the rGO-Al_2_O_3_ nanofluid. As evident in Figure 8a, the worn surface of the steel disk lubricated with base fluid presented with deep furrows and asperities accompanied by dense pits, demonstrating that serious wear occurred. Such phenomenon can be ascribed to the severe abrasive wear and corrosion wear during the sliding process. After the addition of rGO-Al_2_O_3_ nanoparticles to the base fluid, the friction scratches and furrows on the wear scar became shallower with noticeably sparser pits. Meanwhile, large areas of smooth zones were observed on the surface. The flattening and smoothing of the worn surface reflected the polishing effect of nanoparticles [31], especially the high hardness nano-Al_2_O_3_. The surface roughness values along the diameter of the wear track perpendicular to the grinding marks are listed in Table 10. *R*_a_, *R*_p_ and *R*_v_ are, respectively, the average roughness value, the maximum peak height and the maximum valley depth of the contour center line [32]. It is clear that when lubricated with base fluid, serious furrows and scratches were generated on the large area of wear track. In striking contrast, with respect to the lubrication with the optimal 0.20 wt.% nanofluid, the wear track width was significantly reduced. The worn surface became quite smooth, and the *R*_a_, *R*_p_ and *R*_v_ decreased by about 56.0%, 61.1% and 65.3%, respectively. This was due to the deposition and formation of a protective film by the nanoparticles, which effectively isolated the direct contact of friction pairs, and as a result, the asperities and valleys on the worn surface decreased. As a result, the surface quality was improved, and the roughness obviously decreased.

### 3.4. Lubrication Mechanism of rGO-Al_2_O_3_ Nanofluid

**Figure 9:**
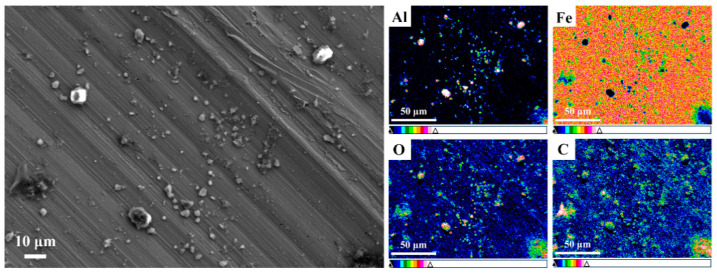
SEM micrograph of worn disk surface lubricated with 0.20 wt.% rGO-Al_2_O_3_ nanofluid and EDS map scanning results.

The typical SEM images and relevant EDS map scanning results of wear track on the disk lubricated with 0.20 wt.% rGO-Al_2_O_3_ nanofluid are shown in Figure 9. It can be seen in Figure 9 that there were plentiful rGO, Al_2_O_3_ and Fe debris adsorbed on the worn surface, which was related to their high surface energy and film-forming capability [33,34]. rGO and Al_2_O_3_ nanoparticles were easy to deposit in furrows to improve the surface quality. This is known by relevant scholars as the mending effect of nanoparticles [35]. Interestingly, the distribution of C and Al elements on the worn surface was rather distinct. It suggests that the majority of the rGO-Al_2_O_3_ nanocomposites were disintegrated during the friction process due to the local high temperature and pressure. It is noteworthy that a small number of large particles (>5 μm in diameter) formed through the soft-agglomeration of nanoparticles due to van der Waals and Coulomb forces [27]. These attractive interaction forces were susceptible to disruption during the friction process, and the effect on the tribological properties of the nanofluid was probably restricted. As the EDS map scanning results in Figure 9 show, a protective tribofilm formed on the friction surface by means of the nanoparticles, and as a result, tribo-oxidation and corrosion were significantly suppressed. In addition, if the sheer force during the friction process is large enough to overcome the van der Waals force, there would be sliding between the rGO layers to further reduce friction force, and this creates the interlayer sliding effect of nanoparticles [36]. Regarding spherical Al_2_O_3_ particles, they can act as bearings between the surface of friction pairs to exhibit antiwear and antifriction effects, which is called the rolling effect [31,37].

**Figure 10:**
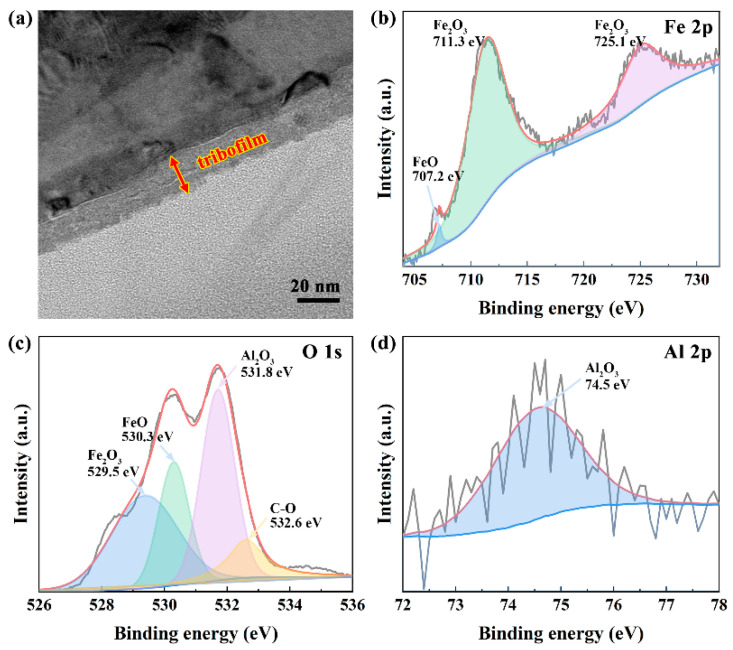
(**a**) TEM image of tribofilm on the worn disk surface, XPS resolved fitting curves of (**b**) Fe 2p, (**c**) O 1s and (**d**) Al 2p spectra of the worn surface lubricated by 0.20 wt.% rGO-Al_2_O_3_ nanofluid.

To provide insights into the lubrication mechanism of the rGO-Al_2_O_3_ nanofluid in the friction process, the worn surface of the steel disk was further characterized using TEM and XPS spectroscopy, as presented in Figure 10. In Figure 10a, the thickness of the tribofilm was about 20 nm, which was formed by tribo-sintering under the joint action of high pressure and frictional heat, thus reducing wear and friction [38]. The specific composition of the tribofilm was investigated using XPS analysis. For the Fe 2p spectrum in Figure 10b, peaks at the binding energy of 707.2 eV, 711.3 eV and 725.1 eV were assigned to the FeO, Fe 2p1/2 and Fe 2p3/2 of Fe_2_O_3_, respectively. The four peaks of O 1s at 529.5 eV, 530.3 eV, 531.8 eV and 532.6 eV were associated with the Fe_2_O_3_, FeO, Al_2_O_3_ and C-O bond. As for the Al 2p spectrum, the binding energy of 74.5 eV was related to Al_2_O_3_. Based on the above results, Fe_2_O_3_ and FeO were generated on the worn surface due to the tribochemical reaction, as described in Equations (6)–(8), while the appearance of Al_2_O_3_ and C-O bond was attributable to the fragmentation of rGO-Al_2_O_3_ nanoparticles during the friction process and their adsorption on the metal surface. It has been revealed that metal oxides contributed to the formation of low-friction tribofilm for excellent self-lubrication and antiwear properties [39]. Combined with the easy-to-shear property of rGO, the lubrication performance of the rGO-Al_2_O_3_ nanofluid was further promoted.

4Fe + 3O_2_ + 6H_2_O = 4Fe(OH)_3_ (6)

2Fe(OH)_3_ = Fe_2_O_3_ + 3H_2_O (7)

2Fe + O_2_ = 2FeO (8)

## 4. Conclusions

The interaction of testing force, rotational speed and nanoparticle concentration on tribological performance was investigated through pin-on-disk experiments via RSM. The lubrication mechanism of rGO-Al_2_O_3_ nanofluid was proposed. The following conclusions were drawn from this study.

(1) According to ANOVA analysis, the quadratic models for all *μ*, *W*_r_ and *R*_a_ values were significant. All three variables were significant for *μ* and *W*_r_ values, while testing force, nanoparticle concentration and its interaction with testing force and rotational speed were identified as significant parameters for *R*_a_ value. The optimized response values according to regression models were 0.088, 2.35 × 10^−7^ mm^3^·N^−1^·m^−1^ and 0.832 μm for *μ*, *W*_r_ and *R*_a_ values. In addition, the experimental results were in good agreement with predicted values.

(2) During pin-on-disk friction tests, 0.20 wt.% rGO-Al_2_O_3_ nanofluid exhibited the optimum lubrication performance. Compared to the base fluid, the *μ*, *W*_r_ and *R*_a_ values were reduced by 45.6%, 90.3% and 56.0%, respectively. The worn surface lubricated by 0.20 wt.% rGO-Al_2_O_3_ nanofluid was smoother and flatter with fewer furrows and scratches. Based on the mathematical model and 3D response results, higher speed and lower force can reduce the friction and wear rate, which provided theoretical guidance for the parameter setting of metal processing in the future.

(3) C, O and Al elements were found on the worn surface, which indicated the formation of tribofilm (~20 nm) due to the tribochemical reaction and physical absorption. The metal oxides (Fe_2_O_3_, FeO and Al_2_O_3_) and nanoparticle debris (Al_2_O_3_ and rGO) were the main components of the tribofilm. The lubrication action of rGO-Al_2_O_3_ nanofluid can be attributed to the polishing effect, mending effect, rolling effect and interlayer sliding effect of nanoparticles.

## Figures and Tables

**Table 1:** Specific parameters of graphene oxide.

Parameters	Value
Appearance	Black powders
Purity	98%
Thickness	5–10 nm
Diameter	3–5 μm
Specific surface area	39.6 m^2^/g
Tap density	0.086 g/cm^3^
Electrical conductivity	600–900 S/cm

**Table 2:** Physicochemical properties of the base fluid.

Parameters	Value
Appearance	Colorless and transparent liquid
Kinematic viscosity (25 °C)	20.9 mm^2^/s
Density	0.9 g/cm^3^
pH value	8.0
Flow point	−10 °C
Defoaming capability	≤2

**Table 3:** Chemical compositions of the pin and disk (wt.%).

C	Si	Mn	S	Cr	Ni	Fe
0.43	0.15	0.48	0.02	0.23	0.26	Balance

**Table 4:** Control factors and the corresponding levels.

Control Factor	Symbol	Units	Level 1	Level 2	Level 3
Force	*F*	N	100	200	300
Speed	*V*	r·min^−1^	200	300	400
Nanoparticle concentration	*N*_c_	wt.%	0.1	0.2	0.3

**Table 5:** Input experiment parameters and response variables.

Test	Input Experiment Parameters			Output Variables		
	*F* (N)	*V* (r·min^−1^)	*N*_c_ (wt.%)	*μ*	*W*_r_ (mm^3^·N^−1^·m^−1^)	*R*_a_ (μm)
1	100	200	0.2	0.153	4.22 × 10^−7^	0.905
2	300	300	0.3	0.158	3.44 × 10^−6^	1.941
3	300	400	0.2	0.088	2.34 × 10^−6^	1.511
4	100	300	0.1	0.203	8.92 × 10^−7^	1.239
5	200	400	0.3	0.163	1.81 × 10^−6^	1.542
6	200	200	0.1	0.224	1.95 × 10^−6^	1.462
7	100	300	0.3	0.179	1.29 × 10^−6^	1.456
8	200	400	0.1	0.168	1.63 × 10^−6^	1.643
9	300	200	0.2	0.142	2.94 × 10^−6^	1.516
10	100	400	0.2	0.124	4.69 × 10^−7^	0.832
11	200	300	0.2	0.128	5.32 × 10^−7^	1.142
12	200	300	0.2	0.137	6.10 × 10^−7^	1.153
13	300	300	0.1	0.174	3.09 × 10^−6^	2.005
14	200	200	0.3	0.182	2.39 × 10^−6^	1.688
15	200	300	0.2	0.124	9.80 × 10^−7^	1.203

**Table 6:** ANOVA table of *μ* value for nanofluids.

Source	Sum of Squares	DF	Mean Square	F-Value	*p*-Value	Remarks
Model	0.0165	9	0.0018	88.07	<0.0001	Significant
A-*F*	0.0012	1	0.0012	56.59	0.0007	Significant
B-*V*	0.0031	1	0.0031	150.14	<0.0001	Significant
C-*N*_c_	0.0009	1	0.0009	45.52	0.0011	Significant
AB	0.0002	1	0.0002	7.52	0.0407	Significant
AC	0.0000	1	0.0000	0.7698	0.4204	
BC	0.0003	1	0.0003	16.47	0.0097	Significant
A^2^	0.0001	1	0.0001	3.34	0.1273	
B^2^	7.41 × 10^−6^	1	7.41 × 10^−6^	0.3565	0.5764	
C^2^	0.0104	1	0.0104	520.18	0.0001	Significant
Residual	0.0001	5	0.0000			
Lack of fit	0.0000	3	5.08 × 10^−6^	0.1147	0.9438	Not significant
Pure error	0.0001	2	0.0000			
Cor total	0.0166	14				

**Table 7:** ANOVA table of *W*_r_ value for nanofluids.

Source	Sum of Squares	DF	Mean Square	F-Value	*p*-Value	Remarks
Model	1.44 × 10^−11^	9	1.60 × 10^−12^	60.08	0.0001	Significant
A-*F*	9.56 × 10^−12^	1	9.56 × 10^−12^	359.76	<0.0001	Significant
B-*V*	2.67 × 10^−13^	1	2.67 × 10^−13^	10.03	0.0249	Significant
C-*N*_c_	2.38 × 10^−13^	1	2.38 × 10^−13^	8.94	0.0304	Significant
AB	1.04 × 10^−13^	1	1.04 × 10^−13^	3.93	0.1044	
AC	6.33 × 10^−16^	1	6.33 × 10^−16^	0.0238	0.8834	
BC	1.80 × 10^−14^	1	1.80 × 10^−14^	0.6760	0.4484	
A^2^	1.06 × 10^−12^	1	1.06 × 10^−12^	40.02	0.0015	Significant
B^2^	3.32 × 10^−13^	1	3.32 × 10^−13^	12.48	0.0167	Significant
C^2^	3.29 × 10^−12^	1	3.24 × 10^−12^	121.88	0.0001	Significant
Residual	1.33 × 10^−13^	5	2.66 × 10^−14^			
Lack of fit	1.80 × 10^−14^	3	6.01 × 10^−15^	0.1046	0.9500	Not significant
Pure error	1.15 × 10^−13^	2	5.74 × 10^−14^			
Cor total	1.45 × 10^−11^	14				

**Table 8:** ANOVA table of *R*_a_ value for nanofluids.

Source	Sum of Squares	DF	Mean Square	F-Value	*p*-Value	Remarks
Model	1.61	9	0.1784	223.13	<0.0001	Significant
A-*F*	0.8071	1	0.8071	1009.30	<0.0001	Significant
B-*V*	0.0002	1	0.0002	0.2890	0.6139	
C-*N*_c_	0.0097	1	0.0097	12.08	0.0177	Significant
AB	0.0012	1	0.0012	1.45	0.2831	
AC	0.0197	1	0.0197	24.69	0.0042	Significant
BC	0.0267	1	0.0267	33.43	0.0022	Significant
A^2^	0.0095	1	0.0095	11.89	0.0183	Significant
B^2^	0.0024	1	0.0024	3.06	0.1406	
C^2^	0.7262	1	0.7262	908.21	<0.0001	Significant
Residual	0.0040	5	0.0008			
Lack of fit	0.0019	3	0.0006	0.5942	0.2655	Not significant
Pure error	0.0021	2	0.0011			
Cor total	1.61	14				

**Table 9:** The predicted and validation results.

Output Variables	Input Parameters			Predicted Result	Desirability	Validation Result
	*F* (N)	*V* (r/min)	*N*_c_ (wt.%)			
*μ*	300	400	0.20	0.089	0.995	0.088
*W*_r_ (mm^3^·N^−1^·m^−1^)	116	354	0.19	2.24 × 10^−7^	1.000	2.35 × 10^−7^
*R*_a_ (μm)	100	400	0.20	0.850	0.984	0.832

**Table 10:** The line roughness of wear track lubricated with base fluid and 0.20 wt.% rGO-Al_2_O_3_ nanofluid.

Lubrication Condition	*R*_a_ (μm)	*R*_p_ (μm)	*R*_v_ (μm)
Base fluid	1.890	4.136	4.655
0.20 wt.% rGO-Al_2_O_3_ nanofluid	0.832	1.610	1.615

## Data Availability

Not applicable.
